# Manual on the proper use of the ^211^At-labeled PSMA ligand ([^211^At]PSMA-5) for clinical trials of targeted alpha therapy (1st edition)

**DOI:** 10.1007/s12149-024-01916-6

**Published:** 2024-03-28

**Authors:** Tadashi Watabe, Masao Namba, Sachiko Yanagida, Yoshihide Nakamura, Takahiro Yamada, Saori Tatsuno, Aritoshi Ri, Shuhei Yoshida, Kumiko Uyama, Seigo Kinuya, Noriyuki Tomiyama, Makoto Hosono

**Affiliations:** 1 Department of Nuclear Medicine and Tracer Kinetics, Graduate School of Medicine, Osaka University, 2-2 Yamada-oka, Suita, Osaka 565-0871 Japan; 2 Japan Radioisotope Association, 2-28-45 Honkomagome, Bunkyo-ku, Tokyo, 113-0021 Japan; 3 Chiyoda Technol Corporation, 1-7-12 Yushima, Bunkyo-ku, Tokyo, 113-8681 Japan; 4 Atomic Energy Research Institute, Kindai University, 3-4-1 Kowakae, Higashi, Osaka 577-8502 Japan; 5 Faculty of Medicine, Kindai University, 377-2 Ohno-Higashi, Osaka-Sayama, Osaka 589-8511 Japan; 6 Japanese Society of Nuclear Medicine, 3-1-17 Nishi-Azabu, Minato-ku, Tokyo, 106-0031 Japan; 7 Department of Radiology, Graduate School of Medicine, Osaka University, 2-2 Yamada-oka, Suita, Osaka 565-0871 Japan

**Keywords:** Astatine, Prostate cancer, Targeted alpha therapy, Radiation exposure, Safety management

## Abstract

Recently, an astatine-labeled prostate-specific membrane antigen (PSMA) ligand ([^211^At]PSMA-5) has been developed for the targeted alpha therapy of patients with prostate cancer. This manual delineates its physicochemical characteristics to assist healthcare professionals in understanding the α-ray-emitting drug of [^211^At]PSMA-5 when administered to patients. The safety considerations regarding the handling and use of this drug in clinical trials are outlined, based on the proper usage manual of previous studies. The dose limits, as defined by the guidelines of the International Commission on Radiological Protection (ICRP) and the International Atomic Energy Agency (IAEA), are assessed for patients’ caregivers and the general public. According to the calculations provided in this manual, clinical trials involving [^211^At]PSMA-5 can be safely conducted for these populations even if patients are released after its administration. Moreover, this manual provides comprehensive guidance on the handling of [^211^At]PSMA-5 for healthcare facilities, and compiles a list of precautionary measures to be distributed among patients and their caregivers. While this manual was created by a research team supported by Ministry of Health, Labour, and Welfare in Japan and approved by Japanese Society of Nuclear Medicine, its applicability extends to healthcare providers in other countries. This manual aims to facilitate conducting clinical trials using [^211^At]PSMA-5 in patients with prostate cancer.

## Purpose

This manual has been created for a clinical trial of an astatine-labeled prostate-specific membrane antigen (PSMA) ligand ([^211^At]PSMA-5) as a treatment for prostate cancer to ensure the safe handling of the drug in compliance with the principles of safety guidelines stipulated by the relevant laws and regulations.

Prostate cancer is the most common cancer in men worldwide. Approximately 1.41 million new cases of prostate cancer are diagnosed in the world according to the Global Cancer Statistics 2020 [1]. Disease progression is relatively slow, but due to the large number of patients, some patients showed progressive courses with multiple metastases or disease recurrence in a relatively short time. Hormonal therapy, including novel androgen receptor pathway inhibitors, and chemotherapy are the primary treatment for advanced castration resistant prostate cancer (CRPC). However, CRPC with multiple metastases has a poor prognosis. PSMA is frequently expressed on the plasma membrane of prostate cancer cells in primary tumors and is especially highly expressed in metastatic lesions.

A lutetium-labeled PSMA ligand ([^177^Lu]PSMA-617), a β-ray-emitting therapeutics, has already been approved in the United States and Europe, and clinical trials are being conducted in Japan based on the proper use manual [2]. [^211^At]PSMA-5 has been developed as a new α-ray-emitting therapeutic agent targeting PSMA and is scheduled to start investigator-initiated clinical trials for the treatment of refractory CRPC. Nuclear medicine therapy utilizes molecular targeted therapy, in which administered radiopharmaceuticals selectively accumulate in lesions such as metastatic tumors scattered in the patient's body. As α-rays deliver large amounts of energy over a short distance of 20–100 µm, they are expected to selectively irradiate cancer cells and produce a significant therapeutic effect with minimal impact on the surrounding tissue.

To safely implement this minimally invasive treatment method, it is essential to take measures to safely handle this radiopharmaceutical to minimize radiation exposure and prevent contamination. Therefore, it is important to ensure that patients, their families, and healthcare personnel fully understand its characteristics. Thus, healthcare professionals who perform this treatment should have a thorough understanding of the physical properties of astatine-211 (^211^At) and the chemical properties of the drug to provide appropriate explanations and guidance to patients. Regarding treatment with ^211^At, investigator-initiated clinical trials using sodium astatide ([^211^At]NaAt) and meta-[^211^At] astato-benzylguanidine ([^211^At]MABG) have already been initiated in Japan, and all involved medical institutions appropriately handle these materials according to the respective manual on proper drug use in clinical trials [3, 4].

In the current manual, we incorporated the intent of the Medical Care Act and recommendations of international organizations on radiation protection [5–8], and hospitals that perform this treatment should follow the radiation safety requirements contained in this manual. This manual was created by a research team supported by Ministry of Health, Labour, and Welfare in Japan and approved by Japanese Society of Nuclear Medicine on December 12, 2023.

## Characteristics of [^211^At]PSMA-5

The physical properties of the nuclide ^211^At are summarized in Table 1. The element At with the atomic number 85 is one of the halogens like iodine. ^211^At has a physical half-life of 7.214 h and emits α-rays. This radionuclide is produced by the ^209^Bi (α, 2n) ^211^At reaction using 30 MeV cyclotron.

**Table 1:** Physical properties of ^211^At

Half-life	Mode of breakdown	Maximum α-ray energy (MeV) and emission rates	Main photon energy (MeV) and emission rates	Emission rate of internal conversion electrons (%)	Effective dose rate constant (μSv・m^2^・MBq^−1^・h^−1^)
7.214 h	α	5.867–41.8%, etc	0.670–0.0035%		0.00580
			0.743–9.5 × 10^−4^%		
Daughter ^207^Bi ^211^Po*	EC	58.20%	0.687–0.26%	0.015	0.00644^a^
			0.0787–31.1% Po-K_α_		
			0.0906–8.5% Po-K_β_		
			0.0124–18.9% Po-L		

^a^Including the contribution from ^211^Po, which is in radiative equilibrium. Values are adapted from the Radioisotope Pocket Data Book (12th Edition), published by the Japan Radioisotope Association, 2020. (EC: electron capture)

The distribution of [^211^At]PSMA-5 in humans was estimated based on the pharmacokinetics of this drug in mice and the pharmacokinetics of the [^18^F]PSMA-1007, a companion diagnostic PET probe targeting PSMA, in humans. Physiological accumulation of [^211^At]PSMA-5 is expected in the kidney and salivary and lacrimal glands as these organs physiologically express PSMA. It may be excreted into the intestinal tract through biliary excretion as well as urinary excretion [9].

## Release of patients after administration of radiopharmaceuticals

### Criteria of release from the radioisotope-controlled area

The release criteria (Notification No. 70 of the Pharmaceutical Safety Bureau) [10] were issued as a guideline to ensure the quality of life of treated patients and the safety of caregivers and the general public from radiation exposure. These release criteria are outlined as follows:

1. Scope of application: Patients who received a radiopharmaceutical leaving a medical radioisotope room or radiotherapy ward in a hospital or returning home after the treatment.
2. Release criteria: As the upper dose limit of 1 mSv per year for the public and 5 mSv per case for caregivers have been defined, taking into consideration the benefit to both patients and caregivers.
3. Precautionary items
  - When the patient leaves the radioisotope-controlled area, written/verbal instructions regarding daily activities, etc. should be given to avoid unnecessary exposure to third parties as much as possible.
  - If the patient has a nursing infant, adequate explanation, attention, and guidance should be given.
  - With regard to protection in accordance with the physical characteristics of radionuclides, explanation to patients and caregivers, and other safety management, the guidelines published by radiation-related academic societies and other organizations should be referred to.

### Factors related to the evaluation of release criteria

The components determining the external dose are contact time with the patient, distance from the patient, and radiation dose rate. Accordingly, the “exposure factor,” which comprises factors to be considered in evaluating the exposure dose of a third party, is set according to the degree of interaction with the patient.

The exposure factor is defined as the ratio of the accumulated dose that third-party individuals would receive to the accumulated dose that these individuals would receive if they stayed at a distance of 1 m from the patient for an infinite time (the time until all nuclides have decayed).

1. Exposure factor for caregivers: 0.5
  Based on actually measured doses to patients who received radiopharmaceuticals, it has been reported that an exposure factor of 0.5 is reasonable for caregivers of patients who require extensive nursing care [14]. In a survey study in Japan that measured the exposure dose from ^131^I-treated patients, an exposure factor of 0.5 was considered appropriate [15]. Based on the above, an exposure factor of 0.5 was adopted for the dose assessment of caregivers after patients left the treating facility.
2. Exposure factor for the public: 0.25
  A publication [14] indicates that the adoption of an exposure factor of 0.25 is appropriate based on actual measured doses to patients’ family members in general households. An exposure factor of 0.25 was adopted for family members other than caregivers and other members of the public after the patient left the treating facility.

## Release of patients who received [^211^At]PSMA-5

### Exposure dose to third parties from patients treated with [^211^At]PSMA-5

The exposure dose to caregivers and the public is determined by external exposure due to radiation emitted from radioactive materials in the body of the treated patient and internal exposure due to contamination of the patient’s excreta. The following is a composite assessment of the doses to which third parties are exposed.

### Evaluation of the external radiation dose

#### Effective dose rate of external exposure at 1 m from patients receiving [^211^At]PSMA-5

The formula for calculating the dose rate of external exposure to a third party from a patient receiving this drug is.

$$I\, = \,A\, \times \,C\, \times \,F_{a} /L.^{2}$$

with.

*I:* Effective dose rate at the calculated evaluation point [μSv h^−1^]

*A*: Residual radioactivity in the body of the treated patient [MBq]

*C:* Effective dose rate constant for ^211^At [μSv m^2^ MBq^−1^ h^−1^]; 0.00644 [μSv m^2^･MBq^−1^ h^−1^] (Table 1)

*F*_*a*_*:* Effective dose transmittance (if there are multiple shields, the total transmittance is the product of the transmittance of each shield)

*L*: Distance from the source to the evaluation point [m]

### Cumulative dose of exposure to third parties from patients treated with [^211^At]PSMA-5

The formula for the cumulative effective dose when a third party is continuously exposed to radiation from a drug-treated patient is.

$$E = A \times \int {(\frac{1}{2}} )^{\frac{t}{T}} dt \times C \times f_{0}$$

with.

*E*: Cumulative effective dose to which a third party is exposed [μSv]

*A*: Residual radioactivity in the body of the treated patient [MBq]

*C:* Effective dose rate constant for ^211^At [μSv m^2^ MBq^−1^ h^−1^]; 0.00644 [μSv m^2^ MBq^−1^ h^−1^] (Table 1)

*T*: Physical half-life of ^211^At

f_0_: Exposure factor (caregiver; 0.5, non-caregiver public; 0.25)

### Factors for assessing cumulative doses of caregivers and the public

1. The calculation of the integrated dose to which a third party is exposed after a patient leaves the treating facility is based on the effective dose rate at a distance of 1 m from the patient’s body surface.
2. The actual radioactivity in the body of a treated patient depends on the physical half-life of ^211^At and the effective half-life of this drug, considering the pharmacokinetics of the drug.
3. Maximum drug dose is defined as 400 MBq to estimate the transition of the ^211^At radioactivity in the body is used to estimate the accumulated dose to which a third party is exposed due to a patient receiving this drug.

### Estimation of cumulative external radiation doses to third parties

The calculation of the effective dose rate of external exposure at a distance of 1 m from the patient’s body surface at a certain time after administration of this drug is as follows: The effective dose rate constant is 0.00644 [μSv・m^2^・MBq^−1^ h^−1^] (Table 1). The effective dose rate at a distance of 1 m from the patient’s body surface immediately after administering this drug at the maximum dose of 400 MBq can be expressed as.

$${4}00\left[ {{\text{MBq}}} \right]\, \times \,0.00{644 }[\mu {\text{Sv}} {\text{m}}^{{2}} {\text{MBq}}^{{ - {1}}} {\text{h}}^{{ - {1}}} \left] {\, \times \,{1 }} \right[{\text{m}}^{{ - {2}}} \left] {\, = \,{2}.{58 }} \right[\mu {\text{Sv}} {\text{h}}^{{ - {1}}} ].$$

Furthermore, as the area under the decay curve to infinity can be calculated using half-life [h]/log_2_, assuming that there is no excretion from the patient, the accumulated effective dose rate at a distance of 1 m from the patient’s body surface until decay to infinity is.

$${2}.{58}\;[\mu {\text{Sv}} {\text{h}}^{{ - {1}}} ]\, \times \,\left( {{7}.{214}/0.{6931}} \right) \, \left[ {\text{h}} \right]\, = \,{26}.{85 }\left[ {\mu {\text{Sv}}} \right]$$

As there is no shielding effect by the patient’s body, the effective dose transmission rate is assumed to be 1. Considering that this drug is administered three times and taking the exposure factor into account, the accumulated doses for caregivers and the public are as follows:

1. Integrated dose for caregivers (exposure factor = 0.5): 26.85 [μSv/treatment] × 3 × 0.5 = 40.28 [μSv/treatment]
2. Total dose to the public (exposure factor = 0.25): 26.85 [µSv/account] × 3 × 0.25 = 20.14 [µSv/account]
3. Both of these values are well below the dose constraint value of 5 mSv (5,000 μSv) per case for caregivers and the annual dose limit of 1 mSv (1,000 μSv) for the general public.

### Evaluation of the internal radiation dose

Excreta from [^211^At]PSMA-5-treated patients may be discharged into rivers via sewage treatment plants and possibly used as drinking water after reprocessing in a safe-side assumption. Therefore, the estimation of internal exposure doses assumes that all of the radioactivity administered to patients will be discharged into rivers.

Regarding the public exposure dose, a study using the Yodo River system model in the Osaka area, which has a high utilization rate of purified and treated water, was conducted in the previous report [14]. As the astatine is a homologous halogen element to the iodine, the exposure dose to caregivers is estimated by referring to the “Evaluation of doses received by caregivers from patients receiving iodine-131” in the “Data concerning the calculation of release criteria” in the Administrative Communication (June 30, 1998) of the Safety Measures Division, Pharmaceutical Safety Bureau, Ministry of Health and Welfare [15]. Based on these study results, the following calculations confirm that the recommendations of the International Commission on Radiological Protection (ICRP) and the safety standards of the International Atomic Energy Agency (IAEA) of 1 mSv (1,000 μSv) annual dose limit for the public and 5 mSv (5,000 μSv) dose constraint per case for caregivers are met.

#### Estimation of the public exposure dose

It is assumed that [^211^At]PSMA-5 treatment will be administered to approximately 10% (1,300 patients) of the 12,759 annual prostate cancer mortalities (as of 2020). Moreover, the population ratio of the Yodo River Basin is estimated to be 0.096 (as of 2020): Yodo River Basin population / total population of Japan =  ~ 12.1 million [16]/ ~ 126.146 million [17] = 0.096. If a dose of 5.0 MBq/kg is administered three times to a patient weighing 80 kg, the total dose is 1,200 MBq/person, and thus the total annual usage in the population of the Yodo River Basin is estimated to be 1.2 × 1,300 × 0.096 ≈ 150 [GBq/year].

To evaluate conservatively assuming that all ^211^At administered to patients flows into the Yodo River system, the radioactivity in the Yodo River system would be 150 [GBq/year]/4.1 [TL/year] ≈ 0.037 [Bq/L]; 4.1 TL is the average annual flow in the Yodo River system (1991–1995) [3].

For the public, the per capita intake of ^211^At per year (assuming a consumption of 2.65 L drinking water per day [18]) is 0.037 [Bq/L] × 2.65 [L/day] × 365 [days/year] ≈ 35.8 [Bq/year]. The annual internal dose in the above case is 35.8 [Bq/year] × 1.1 × 10^−5^ [mSv/Bq] ≈ 0.394 [μSv/year], with 1.1 × 10^−5^ [mSv/Bq] being the effective dose coefficient from oral intake of ^211^At [19]. The calculated dose of 0.394 μSv is much lower than the public annual dose limit of 1 mSv (1,000 μSv).

#### Estimation of caregiver exposure dose

As mentioned above, internal doses for caregivers are estimated by referring to the “Evaluation of doses received by caregivers from patients receiving iodine-131” due to the homology of the halogens astatine and iodine [14].

Based on a report examining air contamination by exhaled breath of patients receiving iodine-131 [20], the maximum volatilization rate of iodine per hour (1.4 × 10^−5^) should also be applied to astatine. The volume of the room with the patient is assumed to be 30 m^3^, the ventilation frequency is assumed to be an average of one time per hour, and the caregiver’s respiratory volume per day is assumed to be 20 m^3^ [14].

The radioactivity ingested by the caregiver per 1 MBq dose is thus.

$${1 }\left[ {{\text{MBq}}} \right]\, \times \,{1}.{4}\, \times \,{1}0^{{ - {5}}} \left[ {{\text{h}}^{{ - {1}}} } \right]\, \times \,\left( {{1}/{3}0 \, \left[ {{\text{m}}^{{ - {3}}} } \right]\, \times \,{1 }\left[ {\text{h}} \right]\, \times \,{2}0 \, \left[ {{\text{m}}^{{3}} /{\text{d}}} \right]\, \times \,{1}/{24 }\left[ {{\text{d}}/{\text{h}}} \right]\, \times \,{1}0.{41 }\left[ {\text{h}} \right]} \right)\, = \,{4}.0{5}\, \times \,{1}0^{{ - {6}}} [{\text{MBq}}]$$

where 10.41 [h] is the average lifetime of ^211^At.

The effective dose of internal exposure due to inhalation ingestion per MBq (applying an exposure factor of 0.5 [14]) is, therefore,

4.05 × 10^−6^ [MBq] × 10^6^ [Bq/MBq] × 2.7 × 10^−5^ [mSv/Bq] × 0.5 = 5.47 × 10^–5^ [mSv] = 0.0547 [μSv]

As the total dose per case with 2.7 × 10^−5^ [mSv/Bq] being the effective dose coefficient of ^211^At due to inhalation [21]. Assuming a maximum dose of 1,200 MBq with three drug administrations to the patient, the caregiver’s internal exposure from inhalation intake would be 0.0547 × 1,200 = 65.64 [μSv].

Adding the internal exposure due to oral intake as calculated in the preceding section for the public, the total internal exposure of caregivers is 65.64 + 0.39 = 66.03 [μSv].

This effective dose of 66.03 μSv is considerably lower than the dose constraint value of 5 mSv (5,000 μSv) per case for caregivers.

### Combined evaluation of external and internal exposure doses

The combined external and internal exposure doses to which caregivers or the public are exposed for a [^211^At]PSMA-5 treatment are shown below.

Caregivers40.28 [μSv] + 66.03 [μSv] = 0.106 [mSv].

Public20.14 [μSv] + 0.39 [μSv] = 0.021 [mSv].

Both values meet the established criteria for the dose limits per person.

### Release of [^211^At]PSMA-5-treated patients from healthcare facilities

When a patient who has just received [^211^At]PSMA-5 leaves a medical radioisotope or radiotherapy room, the recommendations of the ICRP and the safety standards of the IAEA must be met, and the “Guidelines for the release of patients who have received radiopharmaceuticals” (Pharmaceutical Safety Bureau of Japan) [10] must be followed. Its release criteria can be considered to have been satisfied. Therefore, patients receiving [^211^At]PSMA-5 do not require admission to a radiotherapy ward as specified in Article 30–15 of the Enforcement Regulations of the Medical Care Act.

### Precautions for patients and family members

After administration of [^211^At]PSMA-5, traceable amounts of radioactivity are present in body fluids (mainly blood), urine, and feces. As most of this drug is excreted through the kidneys and intestinal tract, patients, family members, and caregivers should be provided with a written briefing explaining the precautions listed below to ensure their understanding prior to drug administration.

#### Precautions during the first 2 days after administration

##### Precautions pertaining to daily activities

1. If bleeding occurs, wipe the blood using toilet paper or other tissues and flush it down the toilet.
2. If there is a possibility of coming into contact with the urine or feces of the patient or touching clothes or other material contaminated with these excreta, rubber disposable gloves should be worn before handling.
3. If body fluids such as the patient’s blood come into contact with the hand or skin, wash this area immediately and thoroughly with soap and water.
4. The patient should abstain from sexual activity.
5. People living with the patient should be separated from the patient as much as possible. The distance should be at least 1 m or 2 m if the patient will be present for a long period. Contact with children and pregnant women should be minimized.
6. The patient should avoid sleeping in the same bed with others. Ensure at least 2 m separation, and sleep in separate rooms if possible.
7. The patient should be the last member of the household to enter the bath. The bathtub should be cleaned thoroughly with detergent using brushes or similar tools after bathing for the day is completed.
8. The patient should refrain from visiting public places (e.g., public transportation, supermarkets, shopping centers, movie theaters, restaurants, sports games) as much as possible.

##### Precautions for handling laundry

1. Clothing worn by the patient should be washed separately from clothes worn by others and should not be washed at the same time. In addition, thoroughly pre-wash sheets and underwear that have been contaminated with the patient’s blood, urine, or other excreta.

#### Precautions regarding urinating, defecating, or vomiting

1. Patients should urinate in a sitting position.
2. If feces or urine drops onto the toilet or floor, wipe it clean with toilet paper or other tissues and flush it down the toilet.
3. Flush the toilet bowl twice after use.
4. Wash your hands thoroughly with soap and water after urinating or defecating.
5. Always wash the hands or skin with soap and water if they come in contact with the patient’s blood or other body fluids, excreta, or vomit.

### Precautions during the first 3 months after administration

Patients of either sex receiving this drug should take measures to prevent a pregnancy.

## Education and training

It is necessary to acquire knowledge about ensuring medical safety and safe handling of radiation related to this treatment. Education and training programs based on this manual shall include the following items.

1. Laws and regulations, notifications, and release criteria related to radiation hazard prevention
2. Chemical and physical properties of this drug and radiation protection
3. Prevention of radiation exposure of healthcare workers and instructions for patients and their families
4. Radiation measurement and safety management of radioactive waste
  Physicians and radiologists who have sufficient knowledge and experience in radiation therapy or who have acquired expertise through education and training conducted in the hospital may play a role as a provider of said therapy, but in such cases, it is desirable that they be appointed by the administrator of the hospital to which they belong or the principal investigator. In addition, a record of the implementation of education and training conducted in the hospital should be prepared. The record of implementation should be retained by the implementing institution for a minimum of 2 years.

## Radiation protection and prevention of radioactive contamination

### Radiation protection practices for the handling of [^211^At]PSMA-5

Procedures for dispensing this drug, handling this drug, and disposal of waste after administration, as well as inspection and decontamination of rooms (including walls, floors, and other surfaces) where this drug has been used, should be conducted appropriately, in reference to the preceding [^211^At]NaAt proper use manual [3].

### Exposure of medical personnel (external and internal exposure)

The maximum dose per treatment dose is 1,200 MBq. Depending on the relationship between the working time and the distance from the source, the external exposure dose for medical personnel is calculated as shown in Table 2.

**Table 2:** External radiation dose for medical personnel

Work phase	Effective dose (per case)			Dose to skin (per case)^a^			Dose limit	
	Working time (min)	Distance (cm)	Exposure dose (mSv)	Working time (min)	Distance (cm)	Radiation dose (mSv)	Effective dose limits (whole body)	Equivalent dose limits (skin)
Preparation	10	50	0.0043	10	10	0.107	Radiological professionals 50 mSv/year	500 mSv/year
Administration	5	50	0.0021	5	5	0.215	100 mSv/5 years Women who may become pregnant 5 mSv/3 months	

^a^Reference values using effective dose rate constants. Measurement of the equivalent dose to the skin shall be performed using the 70-µm dose equivalent

The effective dose *E* [mSv/week] due to internal exposure of workers per week is calculated by the following formula based on the “Notification No. 398 of the Ministry of Health, Labour and Welfare on December 26, 2000” [21].

$$E\, = \,e\, \times \,I$$

where *e* is the effective dose coefficient in case of drug inhalation or ingestion and *I* [Bq] is the quantity of medical radioisotopes ingested by inhalation per week. *I* is calculated by the following formula:

$$I\, = \,{1}.{2}\, \times \,{1}0^{{6}} \, \times \,C\, \times \,t$$

with

1.2 × 10^6^: Volume of air inhaled by an adult in 1 h [cm.^3^/h]

*C*: Average radioactivity concentration in the air per week [Bq/cm^3^].

*t*: Workload [h/week].

and

*C* = *A* × dispersal rate × number of days used per week/(*V* × 10^6^ × 8 [h] × number of days operating exhaust equipment per week)

with

*A*: Maximum expected daily usage quantity [Bq].

*V*: Indoor exhaust volume [m^3^/h]; the system shall be operated for 8 h/day at an exhaust rate of *V*.

For [^211^At]PSMA-5, *A* is 400 MBq, the dispersal rate is 0.001, the daily indoor exhaust volume is 560 [m^3^/h] × 8 [h], days of use per week is 1 day (number of days this drug is used), days of operation of the exhaust system per week is 5 days, operating time is 10 min (0.167 h), and *e* is 2.7 × 10^−5^ [mSv/Bq]. The effective dose *E* [mSv] due to internal exposure per week is thus:

$$C = 400 \, \times \, 10^{6} \times \, 0.001 \, \times \, 1/\left( {560 \, \times \, 10^{6} \times \, 8 \, \times \, 5} \right) = 1.79 \, \times \, 10^{ - 5} \left[ {Bq/cm^{3} } \right]$$

$$I\, = \,{1}.{2}\, \times \,{1}0^{{6}} \, \times \,C\, \times \,0.{167}\, \times \,{1}\, = \,{3}.{58 }[{\text{Bq}}]$$

$$E\, = \,e\, \times \,I\, = \,{2}.{7}\, \times \,{1}0^{{{-}{5}}} \, \times \,{3}.{58}\, = \,{9}.{7}\, \times \,{1}0^{{{-}{5}}} [{\text{mSv}}]$$

This dose is far below the upper dose limit for medical personnel (50 mSv/year) and almost negligible.

## Disposal of medical radioactive materials contaminated by ^211^At

Objects contaminated by this drug are considered medical radioactive contaminated materials. Medical radioactive contaminated materials should be stored in “disposal facilities (storage and disposal facilities)” in hospitals or other healthcare providers and disposed of appropriately through designated contractors.

## Summary

Clinical trials using [^211^At]PSMA-5 can be safely conducted even if patients are released from radioisotope-controlled area immediately after its administration. Moreover, this manual provides detailed guidance on its handling in healthcare facilities, and precautionary measures are also listed for patients and their caregivers. Healthcare professionals should follow the radiation safety requirements outlined in this manual.
